# Mortality Associated with Intensive Care Unit Admission and Mechanical Ventilation in Adults with Acute Chest Syndrome: A Systematic Review

**DOI:** 10.3390/medicina62091731

**Published:** 2026-09-08

**Authors:** Mohammed Essam Shaybah, Osama Alsehli, Ali Al-Harthi, Nada Bajuaifer, Mohammed Bafaqih, Mohammed Alzhrani, Abdulrhman Alasmari, Abdulghafur Kashgari, Omar M. Alhazmi, Anas Sameer Munshi

**Affiliations:** 1 College of Medicine, Umm Al-Qura University, Makkah 24382, Saudi Arabiaalieissaalharthi@outlook.com (A.A.-H.); alhazmii.omar@gmail.com (O.M.A.);; 2 Department of Hematology & Immunology, College of Medicine, Umm Al-Qura University, Makkah 24382, Saudi Arabia

**Keywords:** acute chest syndrome, sickle cell disease, intensive care unit, mechanical ventilation, mortality, systematic review

## Abstract

*Background and Objectives:* Acute chest syndrome (ACS) is the leading cause of sickle cell disease (SCD)-related mortality (~25% of deaths). A subset of patients develops severe respiratory compromise requiring intensive care unit (ICU) admission or mechanical ventilation (MV), a high-risk group with widely variable reported mortality. This review aimed to determine the mortality rates, ICU/hospital length of stay, and reported complications among adult SCD patients with ACS admitted to the ICU or placed on MV. *Materials and Methods:* This PRISMA 2020-compliant systematic review was prospectively registered on PROSPERO (CRD420261295111). Cochrane Library, PubMed, Web of Science, ScienceDirect, EBSCOhost, and Scopus were searched without date restriction. Due to substantial clinical and methodological heterogeneity, the pre-specified meta-analysis was replaced by a narrative synthesis. Methodological quality was assessed using the Newcastle–Ottawa Scale. *Results:* Eight studies were included (one multicenter prospective cohort, two national database studies, three single-center cohorts, and one before–after antimicrobial stewardship study). In-hospital mortality ranged from 0.95% to 3.8%; overall mortality including follow-up reached 12.9% in a dedicated ICU cohort, a distinct endpoint from in-hospital death. Mechanical ventilation was the strongest indicator of mortality, with odds ratios of 67.53 (MV < 96 h) and 8.73 (MV ≥ 96 h) in the largest national cohort. Tricuspid regurgitant jet velocity ≥3 m/second was associated with cor pulmonale, invasive ventilation, and all immediate hospital deaths in one severe cohort. Documented bacterial infection was uncommon (10–20% of episodes), despite frequent antibiotic use, and procalcitonin-guided discontinuation safely reduced antibiotic exposure. All eight included studies were rated high quality on the Newcastle–Ottawa Scale (score ≥ 7/9). *Conclusions:* Mechanical ventilation, pulmonary hypertension/cor pulmonale, and comorbidity burden are the most consistent markers of poor outcome in ACS. The findings support early recognition, severity-based respiratory support, and antimicrobial stewardship, but should be interpreted cautiously given the substitution of narrative synthesis for meta-analysis, heterogeneous populations, and the geographic concentration of the included studies.

## 1. Introduction

Sickle cell disease (SCD) is an inherited blood disorder caused by a missense mutation in the synthesis of hemoglobin, leading to hemoglobinopathy and various complications.

Sickle cell disease prevalence is usually observed in large areas of Sub-Saharan Africa, the Mediterranean basin, the Middle East, and India, and the numbers are expected to increase in both high- and low-income countries over the years, thus making this disease a concern for the foreseeable future [1].

Although the treatment for sickle cell disease has significantly improved throughout the years, from the use of hydroxyurea to more advanced genetic medications that help reduce patients’ mortality and morbidity and increase overall quality of life [2]. Sickle cell disease has multiple complications that need to be addressed. One of the most important ones is acute chest syndrome (ACS), a phenomenon first defined in 1979 by Dr. Samuel Charache, acute infiltration of the chest, supported by X-ray, associated with fever and increased white blood cell counts [3]. Acute chest syndrome results in 25% of sickle cell disease deaths across the globe. This number makes it the leading cause of sickle cell disease complications, leading to mortality [4].

A subset of patients with acute chest syndrome develop a severe respiratory referral or complication requiring the intensive care unit, ICU, and mechanical ventilation. These patients represent a high-risk group that widely varies in mortality in the literature. This variation might be due to the study setting design and institutional resources, and patient demographics across reporting centers. Therefore, a systematic review of data is needed to determine the reported mortality rate among adult sickle cell disease patients with acute chest syndrome admitted to the ICU or placed on mechanical ventilation.

Describe ICU and hospital length of stay across the included studies.

Identify reported complications, including respiratory distress syndrome, septic shock, acute kidney injury, and this population, if mentioned in the studies.

## 2. Materials and Methods

This systematic review was conducted in accordance with the Preferred Reporting Items for Systematic Review and Meta-Analysis (PRISMA 2020) guidelines (Supplementary Materials File S1) [5] and was prospectively registered on PROSPERO (CRD420261295111). The review examines the association between ICU admission and mechanical ventilation with mortality outcomes in adult patients with acute chest syndrome. A meta-analysis was pre-specified in the protocol. However, due to substantial clinical and methodological heterogeneity identified across included studies, a narrative synthesis was performed in place of quantitative pooling. To our knowledge, no prior systematic review has comprehensively examined ICU and mechanical ventilation-associated mortality in this population, identifying a clear gap in the literature that this review addresses.

### 2.1. Eligibility Criteria

Population: Adults aged ≥18 years with sickle cell disease hospitalized with acute chest syndrome.

Exposure: Admission to the intensive care unit (ICU) and/or receipt of mechanical ventilation (invasive or non-invasive).

Primary Outcome: All-cause mortality (in-hospital or short-term mortality as defined by included studies).

Secondary Outcomes: ICU length of stay, duration and type of mechanical ventilation, hospital length of stay, and reported complications such as acute respiratory distress syndrome, septic shock, and acute kidney injury, when available. Additionally, overall ACS-associated mortality across the included study populations is reported where available. Secondary outcomes also included antimicrobial management strategies (e.g., procalcitonin-guided antibiotic discontinuation) where reported by the included studies, given their relevance to ICU-level ACS care.

Study Design: Studies were grouped for synthesis by outcome domain: mortality (primary), and ICU/hospital length of stay, mechanical ventilation duration, and reported complications (secondary). Observational studies (prospective or retrospective cohort and case-control studies) and randomized controlled trials, if identified, were eligible for inclusion.

### 2.2. Search Strategy and Sources

A structured comprehensive search was done across multiple databases, such as Cochrane Library, PubMed, Web of Science, Science Direct, Ebsco Host, and Scopus, to identify relevant studies. No date restrictions were applied, and the search terms combined controlled vocabulary and free-text terms for ‘acute chest syndrome,’ ‘sickle cell disease,’ ‘intensive care,’ and ‘mechanical ventilation’; the full strategy per database is provided in Supplementary Materials File S2.

### 2.3. Study Selection Process

**Figure 1:**
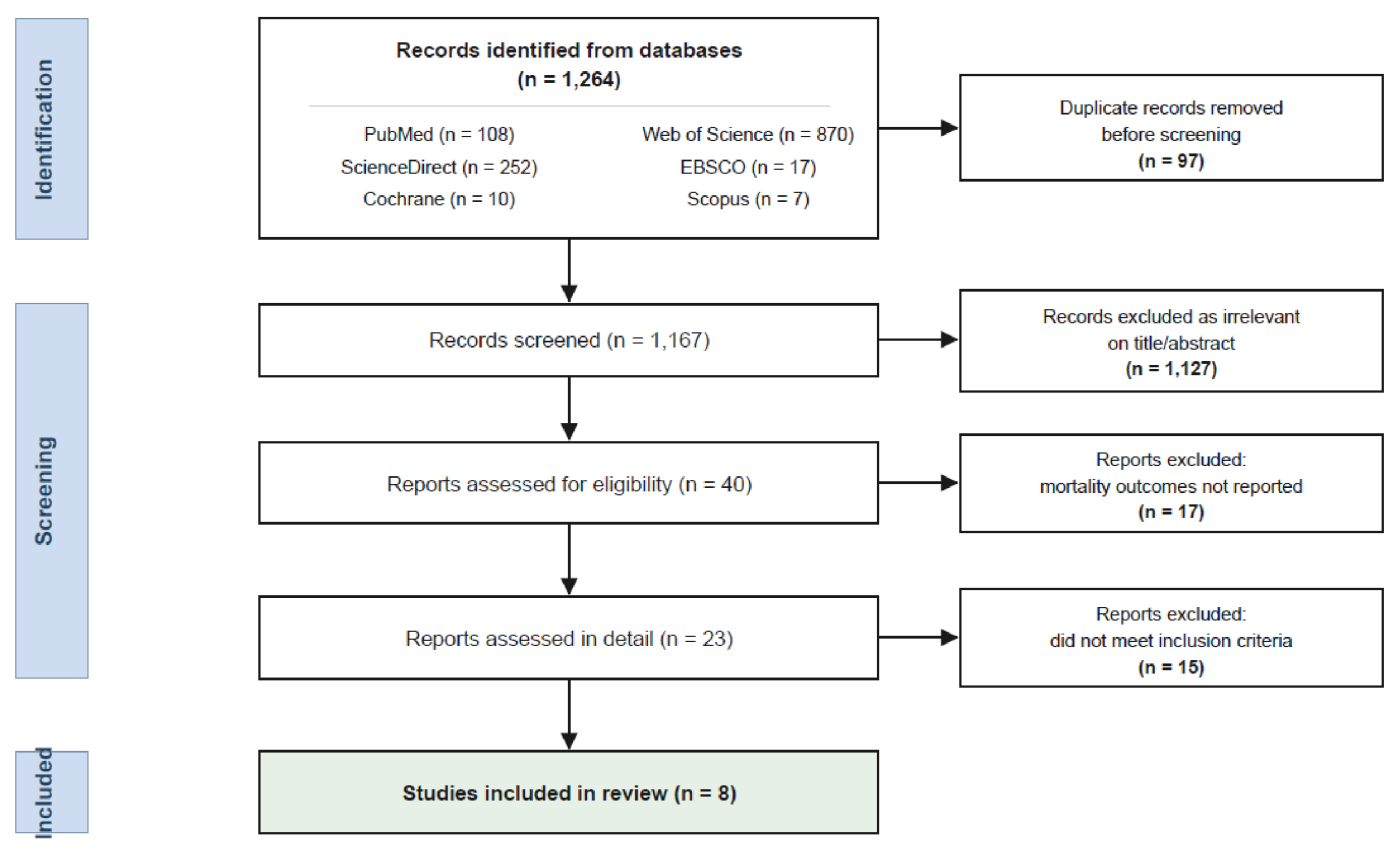
PRISMA 2020 flow diagram.

Three independent reviewers screen titles and abstracts using Rayyan (Rayyan Systems Inc., Cambridge, MA, USA; https://www.rayyan.ai), followed by a full-text review of the eligible citations. Conflict was resolved by discussion or a 4th reviewer; a PRISMA 2020 flow diagram documents the selection process (Figure 1).

Given the narrative-synthesis design and the small number of included studies (k = 8), a formal assessment of reporting bias (e.g., funnel plot or statistical test for small-study effects) was not performed, as such methods require a sufficient number of studies reporting a common effect measure to be reliable. Similarly, certainty of evidence for each outcome was not formally graded using GRADE or an equivalent framework; the Newcastle–Ottawa Scale risk-of-bias ratings served as the primary indicator of study-level methodological quality.

### 2.4. Data Extraction

A standardized data extraction form was developed a priori and piloted on two studies prior to full extraction. The extracted variables included study design, country, study period, sample size, patient demographics (age, sex, sickle cell genotype), ACS diagnostic criteria, ICU and hospital length of stay, mechanical ventilation type and duration, in-hospital and ICU mortality, and reported complications. Extraction was performed independently by two reviewers, with discrepancies resolved by consensus.

The study authors were not contacted for missing or unclear data. Where summary statistics were incompletely reported, this is noted in the corresponding results narrative. All outcome measures and timepoints reported by each study were extracted; no results compatible with the outcome domains were excluded. For studies reporting only aggregate hospitalization-level data (e.g., Allareddy et al.), cell counts below the reporting threshold were estimated from published percentages and total *n* where necessary.

### 2.5. Synthesis

Individual study effect estimates (odds ratios, risk ratios, or hazard ratios, as reported by the original authors) were extracted and presented as reported, without conversion to a common effect measure. Studies were assigned to each synthesis based on whether they reported data for that outcome domain (mortality, length of stay, or complications). Where summary statistics were incompletely reported or denominators differed across studies (e.g., per-episode vs. per-patient), this was noted alongside the relevant result rather than converted to a common denominator. Due to substantial clinical and methodological heterogeneity across the included studies, a narrative synthesis was performed rather than meta-analysis. No pooled estimate was calculated. Quantitative pooling was judged inappropriate due to heterogeneity across several dimensions: different ACS diagnostic definitions, ICU-specific versus general hospital populations, adult-only versus mixed-age cohorts, episode-level versus patient-level units of analysis, and differing mortality time points (in-hospital, 30-day, and follow-up mortality). The findings were descriptively organized by outcome domain. Where mortality rates were reported, they are presented with their original denominators as reported by each study.

## 3. Results

### 3.1. Study Characteristics

Eight studies were included in the narrative synthesis. These comprised one large multicenter prospective cohort, two national administrative database studies, three single-center adult cohort studies, and one prospective before–after antimicrobial stewardship study. Together, the studies represented acute chest syndrome (ACS) episodes or ACS-coded hospitalizations, although quantitative pooling was not performed because the studies differed substantially in population age, severity, setting, outcome definitions, and unit of analysis. The Vichinsky study included a mixed pediatric and adult sickle cell disease (SCD) population across 30 centers, whereas Cheminet, Alsaeed, Mekontso Dessap, Razazi, and Rodriguez focused on adult or adolescent/adult populations. Where age-stratified data were available from Vichinsky et al. (mortality), adult-specific figures are reported alongside whole-cohort figures for comparison. For outcomes not age-stratified in that study (mechanical ventilation rate, transfusion, neurologic complications), whole-cohort figures are used, and this is noted as a limitation. The two largest datasets were national US inpatient analyses by Allareddy et al. and Rodriguez et al. (Table 1).

### 3.2. Patient Characteristics and Clinical Presentation

Across studies, ACS occurred predominantly in patients with severe SCD genotypes, particularly HbSS or S/β0-thalassemia. In the Cheminet cohort, the median age was 27 years, 59% of episodes occurred in males, and 89% of patients had an S/S or S/β0-thalassemia genotype. A high recurrence burden was observed: 81% had a prior history of ACS, with a median of 3 previous episodes. Similarly, Allareddy et al. found that HbSS disease accounted for 84.3% of adult ACS hospitalizations in the US national sample.

Clinical presentation was heterogeneous but commonly included fever, chest pain, respiratory symptoms, and hypoxemia. In the Vichinsky multicenter cohort, nearly half of patients were initially admitted for another indication, mainly pain, and developed ACS subsequently. At ACS diagnosis, patients often had hypoxia, falling hemoglobin, and progressive multilobar pulmonary involvement. In Cheminet et al., fever occurred during 54% of episodes and chest pain in 73%; crackles and bronchial breathing were reported in 64% and 32% of episodes, respectively. Secondary ACS occurred more often than early-onset ACS and had a higher respiratory rate and higher C-reactive protein concentration, although early-onset and secondary ACS did not differ substantially in overall clinical course (Table 2).

### 3.3. Timing of ACS and Recurrence

ACS frequently occurred during hospitalization for vaso-occlusive crisis (VOC). Cheminet et al. classified 44 of 105 episodes as early-onset ACS, occurring within 24 h of admission, and 61 as secondary ACS, occurring more than 24 h after admission. Secondary ACS had a median onset of 2 days after hospital admission. The authors found no major difference in outcomes between early-onset and secondary ACS, although secondary ACS was associated with higher respiratory rate, higher CRP, and more frequent severe tachypnea.

Recurrence was a consistent finding. In Cheminet et al., 26% of ACS episodes were recurrences during the study period, and the authors emphasized that disease-modifying treatment should be reassessed after each ACS episode. In Alsaeed et al., recurrent ACS was reported in 20% of patients, with a 30-day readmission rate of 15%.

### 3.4. Etiology and Microbiological Findings

The included studies consistently showed that ACS has a multifactorial etiology, with both infectious and non-infectious mechanisms. The most extensive etiological evaluation was performed by Vichinsky et al., who identified a specific cause in 38% of all episodes and in 70% of episodes with complete data. Pulmonary fat embolism and infectious pathogens were the major identified causes. Overall, 27 different infectious pathogens were detected, including Chlamydia pneumoniae, Mycoplasma pneumoniae, respiratory syncytial virus, Staphylococcus aureus, Streptococcus pneumoniae, and Haemophilus influenzae.

More recent adult cohorts reported lower microbiological yield. Cheminet et al. found positive microbiological samples in 20% of episodes, but a single bacterial pathogen in only 8.5%, despite antibiotic use in 91% of episodes. Razazi et al. documented proven or possible lung infection in 13% of ACS episodes, with infection absent in 87%. Alsaeed et al. similarly identified pathogens in only 10% of ACS episodes, despite systematic microbiological evaluation. Blood cultures were positive in 6.3%, sputum cultures in 5.0%, and Mycoplasma pneumoniae in 3.8%. These findings indicate that documented bacterial infection is uncommon in modern adult ACS cohorts, although empiric antimicrobial treatment remains widely used because infection cannot be reliably excluded at presentation.

### 3.5. Treatment Strategies

Antibiotic therapy was common across studies, but the stewardship approaches differed. In Alsaeed et al., all ACS episodes were treated with broad-spectrum empirical antimicrobials, most commonly ceftriaxone plus azithromycin in 95% of episodes; 20% required escalation to broader therapy. Cheminet et al. reported antibiotic use in 91% of episodes, despite low bacterial documentation, raising concern for possible antibiotic overuse and highlighting the need for evidence-based antimicrobial stewardship.

Razazi et al. specifically evaluated a procalcitonin-guided strategy to reduce antibiotic exposure. In this before–after study, a protocol recommended stopping antibiotics after three days when bacterial infection was not documented and serial procalcitonin concentrations were below 0.5 µg/L. The intervention increased antibiotic-free days at day 21 from 13 to 15 days and increased the proportion of patients receiving short antibiotic courses of 3 days or less from 9% to 31% without infection relapse or pulmonary superinfection.

Transfusion was also frequently used. Vichinsky et al. reported that phenotypically matched transfusions improved oxygenation and were associated with a 1% alloimmunization rate. Cheminet et al. reported red blood cell transfusion or manual exchange in 50% of episodes, increasing to 67% among severe episodes. In Alsaeed et al., transfusion was used in 50% of ACS episodes, including simple transfusion in 40% and exchange transfusion in 10%. In the severe ICU cohort by Mekontso Dessap et al., the transfusion intensity was higher, with partial exchange transfusion used in 67% of episodes (Table 3).

### 3.6. Clinical Outcomes

Length of stay varied by cohort severity and study setting. Vichinsky et al. reported a mean hospitalization length of 10.5 days. Cheminet et al. reported a median stay of 9 days, while Razazi and Alsaeed reported median stays of 7 days. In the national administrative study by Allareddy et al., the mean length of stay was 7.8 days.

ICU admission and ventilatory support were markers of more severe disease. Cheminet et al. reported ICU transfer in 29% of episodes, although mechanical ventilation was required in only one patient. Alsaeed et al. reported ICU admission in 18.8% and mechanical ventilation in 10% of episodes. In the Vichinsky multicenter cohort, 13% required mechanical ventilation, and 81% of those mechanically ventilated recovered. Allareddy et al. found that 4.6% of national adult ACS hospitalizations required mechanical ventilation, including 2.7% for less than 96 h and 1.9% for 96 h or more.

Mortality ranged from low rates in general adult cohorts to higher rates in severe ICU cohorts. Cheminet et al. reported in-hospital mortality of 0.95%, Razazi et al. 1%, Allareddy et al. 1.6%, Vichinsky et al. 9% (age-stratified adult subgroup, ≥20 years; 3% in the whole cohort including pediatric patients), and Alsaeed et al. 3.8%. The highest mortality was observed in Mekontso Dessap et al.’s severe ACS ICU cohort, where immediate hospital mortality was 5%, and the overall mortality during follow-up was 12.9%. All immediate hospital deaths occurred in patients with tricuspid regurgitant jet velocity of at least 3 m/s (Table 4).

### 3.7. Predictors of Severe Outcomes and Mortality

Several studies identified predictors of severe ACS outcomes. In Vichinsky et al., adults had a more severe course than younger patients, and neurologic events were associated with respiratory failure. The study also identified cardiac disease, multilobar radiographic involvement, and lower platelet count as factors associated with mechanical ventilation or neurologic complications.

Allareddy et al. found mechanical ventilation to be the strongest indicator of in-hospital mortality in adults. Mechanical ventilation for less than 96 h was associated with markedly higher odds of death, and ventilation for 96 h or more also independently increased mortality. In contrast, exchange transfusion was not an independent predictor of mortality, although it was associated with longer hospital stay.

Mekontso Dessap et al. identified acute pulmonary hypertension and right heart strain as important markers of poor outcome in severe ACS. Tricuspid regurgitant jet velocity was at least 3 m/s in 37% of severe ACS episodes, cor pulmonale occurred in 13%, and all invasive ventilation episodes and all immediate hospital deaths occurred in patients with tricuspid regurgitant jet velocity of at least 3 m/s. BNP and cardiac troponin I correlated significantly with pulmonary pressure elevation.

Alsaeed et al. found that ICU admission was associated with higher white blood cell count, lower hemoglobin, higher platelet count, lower oxygen saturation, and higher CRP at presentation. Rodriguez et al. found that asthma did not independently increase inpatient mortality among ACS hospitalizations. Instead, the Charlson Comorbidity Index was the only significant mortality predictor. ACS with asthma was associated with shorter length of stay and lower total hospital cost, without higher rates of transfusion, mechanical ventilation, renal replacement therapy, or hemodialysis for acute kidney injury.

Quality assessment was performed using the Newcastle–Ottawa Scale (NOS). All eight included studies were rated ≥7/9. However, a numerical NOS score should not be equated with high certainty of evidence. The included designs span national administrative database studies, single-center cohorts, and a before–after stewardship study, each susceptible to different forms of confounding, selection bias, and outcome misclassification not fully captured by the NOS instrument (Table 5).

## 4. Discussion

Across the included studies, ACS in SCD was consistently associated with substantial morbidity, frequent hospitalization, high transfusion use, and occasional progression to respiratory failure. The evidence supports ACS as a heterogeneous syndrome rather than a single-pathway disease. Infection and fat embolism were major etiologies when intensive diagnostics were used, but modern adult cohorts found low rates of microbiologically confirmed infection. Mechanical ventilation, pulmonary hypertension, cor pulmonale, hypoxemia, anemia, inflammatory markers, and comorbidity burden emerged as the most consistent markers of poor outcome. Antibiotics were widely used despite low pathogen yield, and procalcitonin-guided discontinuation reduced antibiotic exposure without apparent short-term harm in one before–after study. Overall, the reviewed evidence supports early recognition of ACS during VOC admissions, severity-based transfusion and respiratory support, careful evaluation for cardiopulmonary deterioration, and antimicrobial stewardship when bacterial infection is not documented.

### 4.1. Mortality Burden

The mortality burden of acute chest syndrome (ACS) is not simply a product of respiratory failure alone. Wesevich et al. noted that none of the four deaths in their cohort were directly attributable to identifiable ACS-related respiratory failure on retrospective chart review, suggesting that death in ACS is driven by a cascade of secondary failures rather than a single organ event. This pattern is supported by external evidence. Chaturvedi et al. (2016) found that adults with rapidly progressive ACS far more frequently developed acute kidney injury, hepatic dysfunction, and multiorgan failure (93.8% vs. 10%), with a decline in platelet count at presentation being the only independent predictor [14]. The Cooperative Study of Sickle Cell Disease similarly observed that fatal adult cases generally developed rapid pulmonary failure, with one-third associated with bacteremia [15].

Allareddy et al. reported an overall in-hospital mortality rate of 1.6% (393 deaths) across 24,699 hospitalizations. Mechanical ventilation for fewer than 96 h was associated with dramatically higher odds of death (OR 67.53; 95% CI 34.12–133.63), while ventilation for 96 h or longer was associated with lower, though still significantly elevated, odds (OR 8.73; 95% CI 2.16–35.24). There was also a gradual yearly increase in ACS hospitalizations requiring mechanical ventilation, rising from 2.6% in 2004 to 5.8% in 2010, with mortality the highest in the July–September quarter (2.20%) compared to April–June (1.24%).

Vichinsky et al. reported a 3% in-hospital mortality rate (18 deaths out of 538 patients). Patients aged 20 years or older had a significantly more severe clinical course, with a 9% mortality rate compared to less than 1% in children. The most common causes of death were pulmonary emboli, including bone marrow, fat, or thrombotic emboli (33%), and infectious bronchopneumonia (33%). Infection was a contributing factor in 56% of all deaths.

Alsaeed et al. reported an in-hospital mortality rate of 3.8% (3 deaths), with infectious complications contributing to two of the three fatal outcomes. Cheminet et al. reported a mortality rate of 0.95% (1 death), resulting from multiorgan failure with hepatic sequestration.

Across these studies, in-hospital mortality rates ranged from 0.95% to 3.8%, with overall mortality (including follow-up) reaching 12.9% in the ICU-level cohort of Mekontso Dessap et al.—a distinct, longer-term endpoint rather than a directly comparable in-hospital figure. These deaths were caused by hemodynamic collapse secondary to severe cor pulmonale leading to multiple organ failure.

### 4.2. Mechanical Ventilation as an Indicator of Mortality

Mechanical ventilation (MV) emerged as the most consistent and powerful indicator of in-hospital mortality across the included studies. Allareddy et al. demonstrated that patients requiring MV for fewer than 96 h had an odds ratio of 67.53 for in-hospital death compared to non-ventilated patients, while those requiring MV for 96 h or longer had an odds ratio of 8.73. This estimate derives from a single national administrative dataset and should be interpreted with caution. Mechanical ventilation is largely a marker of ACS severity rather than an independent baseline exposure, and the magnitude of this association likely reflects substantial confounding by indication rather than a causal effect of ventilation itself on mortality. Both associations remained significant after adjusting for patient- and hospital-related factors, including age, sex, race, comorbid burden, and insurance status. This finding is reinforced by the broader critical-care literature. In a meta-analysis of 12,437 ICU patients, invasive mechanical ventilation was the strongest correlate of ICU mortality (pooled OR 16.46; 95% CI 4.37–61.96) [16], and in a dedicated SCD-ICU cohort, acute respiratory distress and kidney injury on admission independently predicted a complicated outcome requiring vital support or death [17].

Vichinsky et al. reported that 13% of all patients in their multicenter cohort required mechanical ventilation, yet 81% of those patients recovered—a rate the authors observed was better than for patients with standard ARDS. Independent predictors of progression to respiratory failure present at diagnosis included multilobar involvement of four or more lobes, a history of cardiac disease, and relative thrombocytopenia with a platelet count below 200,000/mm^3^.

Alsaeed et al. reported that 10% of episodes required mechanical ventilation. Mekontso Dessap et al. found that all five patients who required invasive mechanical ventilation had TRV values of 3 m/s or greater at the time of their ACS episode, underscoring the close relationship between right-sided hemodynamic compromise and the need for ventilatory support.

Regarding the asthma subgroup, Rodriguez et al. found no statistically significant difference in mechanical ventilation use between ACS patients with and without asthma (*p* > 0.05), suggesting asthma does not independently increase the likelihood of requiring MV during an ACS episode.

In the Razazi et al. procalcitonin study, invasive MV was required in 5% of the intervention phase versus 2% of the control phase (*p* = 0.3), and in-hospital death occurred in 3% versus 0% (*p* = 0.4). Both comparisons were underpowered, with only three total MV cases in the entire cohort.

### 4.3. Predictors of ICU Admission

Several studies identified clinical and laboratory predictors of ICU admission. Alsaeed et al. found that ICU patients had significantly higher admission white blood cell counts (18.3 vs. 14.2 × 10^9^/L; *p* = 0.004), lower hemoglobin levels (7.2 vs. 8.1 g/dL; *p* = 0.02), lower oxygen saturation on admission (88.2% vs. 92.5%; *p* = 0.001), and higher C-reactive protein levels (145 vs. 98 mg/L; *p* = 0.008). ICU admission occurred in 18.8% of episodes in that cohort.

Wesevich et al. employed the SpO_2_/FiO_2_ (S/F) ratio as a non-invasive tool to guide clinical decision-making, stratifying patients by home oxygen status. An overall cutoff of S/F < 310 was an independent predictor of ICU transfer within 24 h of ACS diagnosis, with an adjusted odds ratio of 6.52 after adjusting for multiple confounders. Among patients with a diagnostic S/F ratio below 310, 35% were transferred to the ICU within 24 h. Refined cutoffs were S/F <314 for patients not on home oxygen and S/F < 276 for patients on home oxygen. Clinically, the S/F < 310 threshold corresponds to an oxygen saturation of approximately 90% on 2 L/min nasal cannula, making it applicable at the bedside without the need for arterial blood gas sampling. The prognostic validity of the S/F ratio is supported externally by Fukuda et al. (2021), who found it independently associated with ICU and hospital mortality and superior to SAPS II and SOFA [18], and by Catoire et al. (2021), who reported excellent screening performance for hypoxemia in the emergency department (AUC 0.918) [19]. However, Erlebach et al. (2025) caution that the S/F ratio misclassified ARDS severity in 33% of data points and is highly dependent on FiO_2_ settings, limiting its interchangeability with the PaO_2_/FiO_2_ ratio [20]. This remains a single-center validation study, and prospective multicenter testing is required before it can be formally integrated into clinical protocols.

Procalcitonin also showed utility as an ICU admission predictor. Razazi et al. found that episodes with high PCT concentrations had a 62% ICU admission rate compared to 36% in the low-PCT group (*p* = 0.01), alongside higher SOFA scores (4.0 vs. 3.0) and SAPS II scores (18 vs. 10). PCT on Day 1 was notably higher in ICU patients (0.5 µg/L) than in patients in medical wards (0.2 µg/L; *p* = 0.001).

Cheminet et al. reported that 29% of ACS episodes in their cohort led to ICU transfer, though mechanical ventilation was required in only one patient. Secondary ACS cases exhibited a significantly higher median respiratory rate (25 vs. 19.5/min) and higher serum CRP (134.5 vs. 35.2 mg/L) compared to early-onset cases, suggesting greater severity at the time of diagnosis.

### 4.4. Pulmonary Hypertension and Cor Pulmonale During ACS

Mekontso Dessap et al. prospectively evaluated 70 consecutive adults over 84 ACS episodes and found that TRV was less than 2.5 m/s in 40% of episodes, between 2.5 and 2.9 m/s in 23%, and 3 m/s or greater in 37%. These findings extend prior evidence suggesting that a TRV above 2.5 m/s correlates with disease severity. The link between elevated TRV and mortality is well established in the wider SCD literature: Gladwin et al. (2004) reported that a TRV of at least 2.5 m/s carried a markedly increased risk of death (rate ratio 10.1; 95% CI 2.2–47.0) [21], and the walk-PHaSST cohort of 632 patients found a TRV ≥ 3.0 m/s to confer the highest mortality risk of any measured variable (HR 11.1) [22]. It should be noted, however, that this association is not universal. Schimmel et al. (2015) found no significant TRV–mortality relationship in a European cohort, where only NT-proBNP predicted death [23].

Cor pulmonale occurred in 13% (11/84) of episodes, and every patient who developed cor pulmonale had a TRV of 3 m/s or greater. All five patients who required invasive mechanical ventilation and all four patients who died during the immediate hospital course were in this high TRV group, with deaths attributable to hemodynamic collapse secondary to right heart failure leading to multiple organ failure.

Overall mortality in this cohort was 12.9%. Survival was significantly lower in patients whose TRV reached 3 m/s or greater during at least one episode compared to those who did not (*p* < 0.01).

Cardiac biomarkers correlated significantly with TRV. Plasma BNP showed a positive correlation with TRV (rho = 0.54; *p* < 0.01), and a BNP cutoff of 30 pg/mL identified patients with a TRV of 3 m/s or greater with 85% sensitivity and 71% specificity. Cardiac troponin I also correlated with TRV (rho = 0.42; *p* < 0.01), serving as a marker for right ventricular strain and injury.

### 4.5. Infectious Etiology and Antibiotic Management

Pathogen identification rates were consistently low across included studies. Alsaeed et al. identified specific infectious pathogens in only 10% (8/80) of ACS episodes. Blood cultures were positive in 6.3% of cases, identifying Streptococcus pneumoniae, Staphylococcus aureus, and Escherichia coli. Mycoplasma pneumoniae was identified in 3.8% of episodes via PCR. Despite this low identification rate, mortality with infectious complications contributing occurred in two of the three deaths in that cohort.

Vichinsky et al. identified a specific cause in 38% of all episodes and 70% of episodes with complete data. Among infectious pathogens, 27 different organisms were identified, with Chlamydia pneumoniae the most frequent. Other common triggers included Mycoplasma pneumoniae and RSV. Fat embolism, identified through pulmonary macrophages, was also common and frequently co-occurred with an infectious agent.

Cheminet et al. found that only 20% of episodes had a positive microbiological sample, and a single bacterial pathogen was documented in only 8.5% of cases. Yet, 91% of patients received antibiotics. The authors argued this disparity represents significant antibiotic overuse and called for antimicrobial stewardship in ACS.

Vaccination coverage in Alsaeed et al.’s cohort was notably poor, with pneumococcal vaccine uptake at only 50%, annual influenza vaccine at 30%, and COVID-19 vaccine at 40%, highlighting a preventable gap in an already vulnerable population. This deficiency is not unique to that cohort. Keiser et al. (2025) documented comparably low adherence among adults with SCD (pneumococcal 35%, influenza 39%) [24], and Scourfield et al. (2024) emphasized that functional asplenia from early life leaves these patients acutely vulnerable to encapsulated organisms, a risk that established public-health measures such as vaccination can modify [25].

### 4.6. Procalcitonin as a Severity Marker and Stewardship Tool

Razazi et al. performed a before–after study using a PCT-guided algorithm to reduce antibiotic exposure during ACS. The algorithm recommended stopping antibiotics after three days if bacterial infection was not documented and all PCT concentrations were below 0.5 µg/L. In the intervention phase, patients had significantly more days alive without antibiotics at Day 21 compared to the control phase (15 vs. 13 days; *p* = 0.001). The proportion of patients receiving a short antibiotic course of three days or fewer increased from 9% in the control group to 31% in the intervention group (*p* = 0.01), and the median total antibiotic duration fell from 8 to 6 days (*p* < 0.001). There were no instances of infection relapse or pulmonary superinfection in either phase. The safety of PCT-guided antibiotic reduction is corroborated by large pooled analyses. Schuetz et al. (2017) reported lower 30-day mortality and a 2.4-day reduction in antibiotic exposure across 26 randomized trials [26], and Wirz et al. (2018) confirmed a survival benefit and shorter treatment duration in ICU patients [27]. Conversely, the pragmatic ProACT trial (Huang et al., 2018) found that providing PCT results did not reduce antibiotic use compared with usual care, underscoring that real-world implementation and protocol adherence remain key limitations [28].

Beyond its stewardship role, PCT functioned as a severity stratifier. Higher PCT concentrations on Day 1 were associated with significantly worse inflammatory markers (fever 92% vs. 58%; WBC 17.2 vs. 13.2 × 10^9^/L; CRP 174 vs. 81 mg/L), greater hemolysis (LDH 619 vs. 466 IU/L), and higher organ dysfunction scores (SOFA 4.0 vs. 3.0; SAPS II 18 vs. 10), even though only 13% of all episodes had documented infection. This supports PCT as a composite signal of inflammatory, hemolytic, and organ dysfunction severity in SCD-ACS rather than a marker of bacterial infection alone.

Analysis of protocol adherence within the intervention phase found that clinicians who prolonged antibiotics beyond the algorithm’s recommendation had patients with lower oxygen saturation (95% vs. 98%; *p* = 0.006) and a higher rehospitalization rate (17% vs. 0%; *p* = 0.02). These findings suggest that clinicians substituted clinical gestalt based on SpO_2_ for PCT-guided decision-making and that this deviation was paradoxically associated with worse downstream outcomes.

Pending further validation, PCT-guided discontinuation may be considered in hemodynamically stable ACS patients with declining PCT trends, mirroring the protocols validated in ProACT and Schuetz et al. trials. However, ProACT’s neutral real-world result underscores that implementation fidelity—not just protocol design—determines whether stewardship benefits materialize.

### 4.7. Comorbidities, Genotype, and Independent Predictors of Mortality

Allareddy et al. found that the HbSS genotype, present in 84.3% of hospitalizations, did not independently influence in-hospital mortality or length of stay. After adjusting for mechanical ventilation status, the overall comorbid burden also did not influence the mortality rate (*p* = 0.11). Exchange transfusion was not an independent predictor of in-hospital mortality (*p* = 0.07), though it was associated with a 40% increase in length of hospital stay.

Rodriguez et al. reached a different conclusion regarding comorbidity. Using the Charlson Comorbidity Index (CCI), they found that, for every one-unit increase in CCI score, the odds of in-hospital mortality increased by 52% (aOR 1.52; *p* < 0.001). CCI was the only independent predictor of mortality identified in that analysis.

In the asthma subgroup analysis, Rodriguez et al. found that ACS patients with asthma did not have higher odds of death compared to those without asthma (*p* = 0.176) [9]. This contrasts with older data from the Cooperative Study of Sickle Cell Disease, which suggested asthma increased the risk of death by 2.36 times. Boyd et al. (2007) reported a more than two-fold higher all-cause mortality risk in patients with sickle cell anemia and asthma (HR 2.36; 95% CI 1.21–4.62) [29], illustrating that the prognostic role of asthma in this population remains contested. Patients with asthma had a shorter length of stay (coefficient −0.65; 95% CI −1.14 to −0.15; *p* = 0.009), which the authors attributed in part to the younger age of asthma patients, supported by a high correlation with CCI. There was also a significant association between COPD and the asthma cohort (*p* < 0.001), with the authors proposing that repetitive ACS episodes and asthma cause structural lung changes that may progress to COPD-like conditions. Asthma was more highly correlated with thalassemia S and HbSS genotype than with HbSC.

Vichinsky et al. identified neurological complications as an additional mortality-associated outcome. Neurologic events occurred in 11% of patients, and among those, 46% developed respiratory failure and 23% died. Relative thrombocytopenia below 200,000/mm^3^ at diagnosis was the only independent predictor of neurological complications. The prognostic weight of early thrombocytopenia is echoed externally by Chaturvedi et al. (2016), in whom a decline in platelet count at presentation was the sole independent predictor of rapidly progressive ACS (OR 4.82; 95% CI 1.20–19.39) [14].

### 4.8. Early-Onset Versus Secondary ACS and Treatment Adherence

Cheminet et al. examined the relationship between the timing of ACS onset and clinical outcomes. Of their episodes, 42% were early-onset and 58% were secondary, with secondary cases having a median onset of 2 days after admission. Secondary ACS cases showed significantly higher respiratory rates (25 vs. 19.5/min) and higher CRP (134.5 vs. 35.2 mg/L), though the authors noted CRP elevation may reflect the normal trajectory of a non-complicated vaso-occlusive crisis.

Despite these differences, the authors concluded that secondary and early-onset ACS do not differ significantly overall, suggesting they may share similar pathophysiological mechanisms. The median hospital stay was 9 days, nearly double the center’s average for a non-complicated vaso-occlusive crisis.

Treatment adherence was notably poor in this cohort. Only 61% of patients were taking disease-modifying therapy at the time of admission, with 94% of those on hydroxyurea, despite having a prior history of ACS. The authors recommended that disease-modifying treatments be systematically reassessed after every ACS episode.

### 4.9. Additional Descriptive Outcomes

Vichinsky et al. reported that 72% of patients received transfusions, with both simple and exchange transfusions resulting in significant improvements in oxygenation (mean SaO_2_ increased from 91% to 94%). Bronchodilators were used in 61% of patients, with 20% of those treated showing clinical improvement defined as a 15% or greater increase in FEV_1_. The mean duration of hospitalization was 10.5 days.

Alsaeed et al. reported a median length of stay of 7 days (IQR 5–11) and a 30-day readmission rate of 15%. Recurrent ACS was observed in 20% of patients within the follow-up period.

Allareddy et al. found that length of stay was significantly prolonged by mechanical ventilation for 96 h or more, exchange transfusion, and increased comorbid burden. The study’s unit of analysis was hospitalizations rather than individual patients, meaning readmissions could not be identified—a limitation the authors acknowledged.

### 4.10. Limitations

This review has several limitations. The evidence base is characterized by substantial clinical and methodological heterogeneity—differing populations (pediatric-inclusive vs. adult-only), settings, outcome definitions, and units of analysis (hospitalizations vs. patients)—which precluded meta-analytic pooling and limits the precision of any single mortality estimate. Most included studies were retrospective, and one relied on administrative/coded data subject to misclassification. Included studies were geographically concentrated, limiting generalizability, including one included study (Vichinsky et al.) that enrolled a mixed pediatric–adult population. Mortality was age-stratified, and adult-specific figures are used where cited. But other outcomes from that study (mechanical ventilation rate, transfusion, neurologic complications) reflect the whole cohort, including pediatric patients, since the original study did not report these outcomes by age group. Additionally, the study authors were not contacted for missing or unclear data, which may have limited the completeness of extraction, particularly given the small number of included studies (k = 8) and incomplete reporting of several outcomes.

Regarding the review process, the substitution of narrative synthesis for the pre-specified meta-analysis, while justified by heterogeneity, reduces the quantitative precision of our conclusions relative to the original protocol. The search relied on six databases without a dedicated grey-literature or trial-registry search, and conference abstracts without full-text data were excluded, which may have omitted eligible unpublished findings. No language restriction was explicitly pre-specified, which should be clarified for reproducibility.

### 4.11. Future Recommendation

Future prospective, multicenter studies with harmonized ICU-admission and ventilation criteria are needed to validate these mortality predictors and support development of a dedicated ACS-ICU severity score.

## 5. Conclusions

Mechanical ventilation, pulmonary hypertension/cor pulmonale, and comorbidity burden are the most consistent markers of poor outcome in ACS. The findings support early recognition, severity-based respiratory support, and antimicrobial stewardship in this population.

## Figures and Tables

**Table 1:** Study Characteristic.

Study ID	Sub-Group	Mean Age (SD)	Female Sex	SCD Type (Hb SS)	Normal (18.5–24.9)	History of Asthma	Prior ACS	Home Oxygen Use	Baseline Hemoglobin (g/dL)	Platelets (×10^3^/µL) at Diagnosis	Average Hospital Stay (LOS)
Wesevich et al. (2025) [6]	No ICU Transfer (*n* = 175)	28.7 years (9.7)	93 (53%)	123 (70%)	121 (69%)	68 (39%)	146 (83%)	26 (15%)	7.7 (1.5)	369 (164)	8.5 days
	ICU Transfer (*n* = 52)	29.7 years (10.0)	30 (58%)	36 (69%)	23 (44%)	24 (47%)	47 (90%)	17 (33%)	7.2 (1.5)	284 (136)	12.0 days
	*p*-value	0.59	0.5	0.34	0.01	0.33	0.36	0.05	0.12	0.002	0.025
Study 2: Cheminet et al. (2023) [7]	Total Cohort (*n* = 105)	27 [22–34] years	43 (41%)	92 (88%)	21.8 [19.5–23.9]	14 (13%)	66 (81% of patients)	N/A	8.2 [7–9.5]	352 [253–445.5]	9 [7,8,9,10,11] days
Study 3: Allareddy et al. (2014) [8]	Total Cohort (*n* = 24,699)	33.2 (0.19) years	12,696 (51.4%)	20,814 (84.3%)	N/A	3546 (14.3%)	N/A	N/A	N/A	7.8 days (Mean)	N/A
Study 4: Rodriguez et al. (2025). [9]	Total Cohort (*n* = 26,280)	31 years	12,809 (48.74%)	23,035 (93% of SS/SC)	N/A	5685 (21.64%)	N/A	N/A	N/A	6.9 days (Mean)	N/A
Mekontso Dessap et al. (2008) [10]	ICU: Low TRV (*n* = 53 episodes)	24 (21–33) years	22 (41.5%)	52 (98%)	21.7 (19.3–23.7)	N/A	44 (83%)	10 (19%)	8.8 (7.5–10.4)	350 (246–492)	11.0 (9.0–15.0) days
	ICU: high TRV (*n* = 31 episodes)	23 (22–34) years	15 (48.4%)	31 (100%)	22.6 (20.6–24.2)	N/A	23 (74%)	6 (19%)	8.1 (6.8–9.8)	312 (191–393)	11.0 (7.0–18.0) days
Vichinsky 2000 [11]	Total Cohort (*n* = 671)	13.8 years	282 (42%)	550 (82%)	N/A	74 (11%)	450 (67%)	N/A	7.7	23,000	10.5 days
Alsaeed et al. (2025) [12]	(*n* = 60 patients)	24.6 ± 8.3 years	25 (41.7%) [Calculated: 35/60 were male]	52 (86.7%)	N/A	25% used bronchodilators	20 (33.3%) [≥2 episodes during 5-yr study]	N/A	9.1 ± 1.3 g/dL (steady state)	425 ± 145	7 days (IQR: 5–11)
Razazi et al. (2020) [13] (*n* = 100 patients)	(*n* = 100 patients)	N/A	N/A	N/A	N/A	N/A	N/A	N/A	N/A	352 [253–445.5]	7 days (IQR: 6–12)

N/A: not reported by the original study.

**Table 2:** Clinical Presentations.

Study	Age/Sex/Genotype	ACS Timing or Clinical Setting	Main Clinical Features	Microbiology/Etiology
Vichinsky et al., 2000 [11]	Mean age at first ACS episode 13.8 years; 58% male; 82% HbSS	52% admitted with ACS; 48% admitted for another reason, mainly pain; ACS developed after a mean of 2.5 days among those initially admitted for other reasons	Fever 80%, cough 62%, chest pain 44%, tachypnea 45%, shortness of breath 41%; hypoxia, falling hemoglobin, and multilobar involvement were common	Specific cause identified in 38% of all episodes and 70% of episodes with complete data; causes included pulmonary fat embolism and 27 infectious pathogens.
Mekontso Dessap et al., 2008 [10]	70 adults; 68 HbSS, 1 HbSC, 1 HbS-β+ thalassemia	Severe ACS requiring ICU admission	Chest pain 95%, fever 57%, cough 63%, VOC during severe ACS 80%; bilateral radiographic involvement 76%	Focus was cardiopulmonary physiology rather than pathogen identification; TRV ≥ 3 m/s occurred in 37% of episodes and cor pulmonale in 13%.
Allareddy et al., 2014 [8]	Mean age 33.2 years; 48.6% male; HbSS in 84.3% of hospitalizations	Adult ACS hospitalizations identified by ICD-9 codes in NIS	63.7% had ≥1 comorbid condition; 95.5% were emergency/urgent admissions	Microbiology not available in administrative database.
Razazi et al., 2020 [13]	Adults with genetically proven SCD; detailed baseline characteristics not central to the main comparison	ACS managed in medical wards or ICU	Study focused on antibiotic exposure and PCT-guided discontinuation	Possible or proven lung infection diagnosed in 13% of all ACS episodes; PCT < 0.5 µg/L on days 1–3 in 64% of patients.
Cheminet et al. (2023) [7]	Median age 27 years; 59% male; 89% S/S or S/β0-thalassemia; 81% had previous ACS	44/105 episodes early-onset ≤24 h; 61/105 secondary >24 h; secondary ACS median onset 2 days after admission	Fever 54%, chest pain 73%, crackles 64%, bronchial breathing 32%; recurrence during study in 26% of episodes	Positive microbiological test in 20%; single bacterial pathogen in 8.5%; infectious triggers considered uncommon.
Rodriguez et al., 2025 [9]	Mean age 31 years overall; ACS with asthma group younger than non-asthma group, 28 vs. 32 years; 91.84% Black	ACS hospitalizations divided into asthma vs. no asthma cohorts	Asthma group represented 21.64% of hospitalizations; COPD and cigarette use were more common in asthma cohort	Microbiology not available in administrative database.
Alsaeed et al. (2025) [12]	Mean age 24.6 ± 8.3 years; 58.3% male; 86.7% HbSS	ACS was reason for admission in 75%; developed during hospitalization in 25%; concurrent VOC in 90%	Chest pain 80%, shortness of breath 75%, fever 60%, cough 55%, tachypnea 70%, O2 saturation <92% in 50%; unilateral infiltrate 70%	Pathogen identified in 10%; blood culture positive 6.3%, sputum culture 5.0%, *Mycoplasma pneumoniae* 3.8%.
Wesevich et al. (2025) [6]	Mean age 28.9 years, SD 9.7; 54% female; 100% non-Hispanic Black. HbSS was present in 70% of hospitalizations; other genotypes included HbSC, HbSβ+, and HbSβ0.	Prior VOC was reported in 202/227 hospitalizations, 89%; prior ACS in 193/227, 85%; asthma in 92/227, 41%; home oxygen in 43/227, 19%; hydroxyurea in 122/227, 54%.	ACS was defined by at least one symptom—fever, chest pain, hypoxemia, cough, wheezing, or shortness of breath—plus a new opacity on chest radiography. Individual symptom frequencies were not reported.	At ACS diagnosis, chest imaging showed 1 opacified lobe in 114 hospitalizations, 50%; 2 lobes in 91, 40%; and ≥3 lobes in 22, 10%. Platelet count was lower in ICU-transfer hospitalizations than in non-ICU cases, 284 vs. 369 × 10^3^/µL, *p* = 0.002. Nasal viral positivity was 11% in both ICU and non-ICU groups. Among 79 hospitalizations with COVID-19 status assessed, 3 had current infection and 4 had prior infection.

**Table 3:** Management Strategy.

Study	Antibiotic Management	Transfusion/Exchange Transfusion	Respiratory Support/ICU Care	Other Management
Vichinsky et al., 2000 [11]	All patients received antibiotics; protocol used cephalosporin plus erythromycin for 7–10 days	Phenotypically matched transfusions improved oxygenation; alloimmunization rate 1%	13% required MV; 81% of ventilated patients recovered	Bronchodilators used for reactive airway disease; 20% of treated patients had clinical improvement; incentive spirometry and standardized monitoring were included.
Mekontso Dessap et al., 2008 [10]	Antibiotics used in 76/84 episodes, 92%	Simple transfusion in 14/84, 17%; partial-exchange transfusion in 56/84, 67%; median 3 RBC units	Noninvasive MV > 4 h in 9/84, 11%; invasive MV in 5/84, 6%; all patients were ICU cases	Echocardiography and biomarkers were used to assess pulmonary hypertension and right-heart strain.
Allareddy et al., 2014 [8]	Not reported in NIS	ET in 1489/24,699 hospitalizations, 6%	MV < 96 h in 665/24,699, 2.7%; MV ≥ 96 h in 468/24,699, 1.9%	Study adjusted for patient and hospital factors in mortality and LOS models.
Razazi et al., 2020 [13]	PCT algorithm recommended stopping antibiotics after 3 days if no bacterial infection and serial PCT <0.5 µg/L; intervention increased antibiotic-free days	NR	ICU admission in 47/103 episodes, 46%	PCT-guided strategy increased short antibiotic courses ≤ 3 days from 9% to 31%.
Cheminet et al., 2023 [7]	Antibiotics in 96/105 episodes, 91%	Transfusion in 53/105, 50%; manual blood exchange in 27/53 transfused episodes; erythrocytapheresis in 3/53	ICU transfer in 30/105, 29%; artificial ventilation in 6/30 ICU transfers	Authors highlighted possible antibiotic overuse because bacterial documentation was low.
Rodriguez et al., 2025 [9]	Not reported in NIS	Transfusion rates did not differ significantly between ACS with asthma and ACS without asthma; simple transfusion similar between groups	MV rates did not differ significantly between asthma and non-asthma cohorts	Study compared asthma vs. non-asthma groups for LOS, cost, transfusion, MV, CRRT, and dialysis for AKI.
Alsaeed et al., 2025 [12]	Ceftriaxone plus azithromycin in 76/80 episodes, 95%; escalation in 16/80, 20%	Transfusion in 40/80, 50%; simple transfusion 32/80, 40%; exchange transfusion 8/80, 10%	Supplemental oxygen 44/80, 55%; noninvasive ventilation 5/80, 6.3%; ICU admission 15/80, 18.8%; MV 8/80, 10%	Bronchodilators in 20/80, 25%; all patients received pain management and incentive spirometry.
Wesevich et al., 2025 [6]	ACS-directed antibiotics were used in nearly all hospitalizations: 172/175, 98%, without ICU transfer and 52/52, 100%, with ICU transfer. Antibiotic initiation was also one of the three key time points used to calculate SaO_2_/FiO_2_ ratio.	Overall, 45/227 hospitalizations, 20%, had no transfusion; 137/227, 60%, received simple transfusion only; and 45/227, 20%, received RCE. RCE was much more common in ICU-transfer hospitalizations than non-ICU hospitalizations, 67% vs. 6%, *p* < 0.001. Transfusion decisions were made according to clinician judgment.	ICU transfer occurred in 52/227 hospitalizations, 23%. The study did not report separate rates of mechanical ventilation, noninvasive ventilation, or high-flow nasal cannula use as clinical outcomes. All patients who died in hospital had been transferred to ICU, so the composite endpoint of ICU transfer or death was equivalent to ICU transfer.	SaO_2_/FiO_2_ was calculated from pulse oximetry SpO_2_ and oxygen delivery data from ED presentation through 96 h after ACS diagnosis. ACS severity was evaluated using three proxies: number of opacified lobes, RCE use, and ICU transfer. The authors proposed SaO_2_/FiO_2_ cutoffs of <310 for all patients, <314 for those without home oxygen, and <276 for those with home oxygen to guide higher-level care decisions.

**Table 4:** Study Summary.

Vichinsky et al., 2000 [11]	ACS is commonly precipitated by infection and pulmonary fat embolism. Older patients and those with neurologic symptoms are at higher risk for respiratory failure, but most ventilated patients recover with aggressive treatment.
Mekontso Dessap et al., 2008 [10]	Pulmonary pressure elevation is common during severe ACS and is associated with cardiac biomarker elevation, cor pulmonale, invasive ventilation, and increased mortality.
Allareddy et al., 2014 [8]	In a large national adult cohort, MV was the major indicator of inpatient mortality and resource use; ET was associated with longer LOS but not independently with mortality.
Razazi et al., 2020 [13]	PCT-guided antibiotic discontinuation reduced antibiotic exposure during ACS without infection relapse or pulmonary superinfection in this before-after study.
Cheminet et al., 2023 [7]	Most ACS episodes occurred after admission for VOC, but early-onset and secondary ACS did not differ significantly. Microbiologically proven infection was uncommon despite frequent antibiotic use.
Rodriguez et al., 2025 [9]	Asthma did not independently increase inpatient mortality or major inpatient complications among ACS hospitalizations; overall comorbidity burden was more important.
Alsaeed et al., 2025 [12]	Pathogen identification was low despite systematic microbiological workup. Broad empirical coverage was commonly used, but suboptimal pneumococcal vaccination highlighted an important prevention gap.
Wesevich et al., 2025 [6]	The study concluded that bedside SaO_2_/FiO_2_ ratio is a simple, inexpensive, and noninvasive marker associated with ACS severity, RCE use, radiographic extent, and ICU transfer and should be prospectively evaluated as a triage tool for adult SCD patients hospitalized with ACS.

**Table 5:** Newcastle–Ottawa Scale (NOS). ★ indicates the criterion was met; ○ indicates the criterion was not met.

	Selection (max 4 ★)				Comparability (max 2 ★)		Outcome (max 3 ★)			Total ★/9	Quality Grade
Study (First Author)	1. Representativeness of Exposed Cohort	2. Selection of Non-Exposed Cohort	3. Ascertainment of Exposure	4. Outcome Not Present at Start of Study	5. Comparability: Main Factor	6. Comparability: Additional Factor	7. Assessment of Outcome	8. Follow-up Duration Adequate	9. Adequacy of Follow-Up		High ≥ 7 Mod 5–6 Low ≤ 4
**Cheminet 2023** [7]	**★**	**★**	**★**	○	**★**	**★**	**★**	**★**	**★**	**8**	**High** **(8/9)**
**Vichinsky 2000** [11]	**★**	**★**	**★**	**★**	**★**	**★**	**★**	**★**	**★**	**9**	**High** **(9/9)**
**Alsaeed 2025** [12]	**★**	**★**	**★**	○	**★**	○	**★**	**★**	**★**	**7**	**High** **(7/9)**
**Allareddy 2014** [8]	**★**	**★**	**★**	○	**★**	**★**	**★**	**★**	**★**	**8**	**High** **(8/9)**
**Razazi 2020** [13]	**★**	**★**	**★**	○	**★**	○	**★**	**★**	**★**	**7**	**High** **(7/9)**
**Mekontso Dessap 2008** [10]	**★**	**★**	**★**	**★**	**★**	○	**★**	**★**	**★**	**8**	**High** **(8/9)**
**Guevara Rodriguez 2025** [9]	**★**	**★**	**★**	○	**★**	**★**	**★**	**★**	○	**7**	**High** **(7/9)**
**Wesevich 2025** [6]	**★**	**★**	**★**	○	**★**	○	**★**	**★**	**★**	**7**	**High** **(7/9)**

## Data Availability

The original contributions presented in this study are included in the article. Further inquiries can be directed to the corresponding author.

## Supplementary Materials

The following supporting information can be downloaded at https://www.mdpi.com/article/10.3390/medicina62091731/s1. File S1: PRISMA 2020 Checklist; File S2: Database Search Strategy.
