# Supplementary material for: Mortality Associated with Intensive Care Unit Admission and Mechanical Ventilation in Adults with Acute Chest Syndrome: A Systematic Review
Source: Medicina (Kaunas). 2026 Sep 8;62(9):1731. doi: 10.3390/medicina62091731 (PMC13609638; doi:10.3390/medicina62091731)
Supplement: Supplementary file 1 [file medicina-62-01731-s001.zip › Supplementary Materials File S1.pdf]

PRISMA 2020 Checklist

Section and Topic	Item #	Checklist item	Location where item is reported
**TITLE**			
Title	1	Identify the report as a systematic review.	Title page — "...A Systematic Review".
**ABSTRACT**			
Abstract	2	See the PRISMA 2020 for Abstracts checklist.	Abstract (Background/Objective/Methods/Results/Conclusion/Keywords).
**INTRODUCTION**			
Rationale	3	Describe the rationale for the review in the context of existing knowledge.	Introduction, paragraphs 1-3.
Objectives	4	Provide an explicit statement of the objective(s) or question(s) the review addresses.	Introduction, final paragraph; Abstract, Objective.
**METHODS**			
Eligibility criteria	5	Specify the inclusion and exclusion criteria for the review and how studies were grouped for the syntheses.	Methods, "Eligibility Criteria" (Population, Exposure, Primary/Secondary Outcomes); Study Design paragraph now states studies were grouped for synthesis by outcome domain (mortality primary; LOS, MV duration, complications secondary).
Information sources	6	Specify all databases, registers, websites, organisations, reference lists and other sources searched or consulted to identify studies. Specify the date when each source was last searched or consulted.	Methods, "Search strategy and sources" (Cochrane Library, PubMed, Web of Science, ScienceDirect, EBSCOhost, Scopus; no date restrictions applied). Not reported: the date each database was last searched is still not stated.
Search strategy	7	Present the full search strategies for all databases, registers and websites, including any filters and limits used.	Methods states the search string is "provided in Supplementary Appendix 1." Not verifiable here — confirm Appendix 1 is attached to the submission with the actual search strings.
Selection process	8	Specify the methods used to decide whether a study met the inclusion criteria of the review, including how many reviewers screened each record and each report retrieved, whether they worked independently, and if applicable, details of automation tools used in the process.	Methods, "Study selection process" (three independent reviewers, Rayyan AI, conflicts resolved by discussion or 4th reviewer, PRISMA flow diagram Fig. 1).
Data collection process	9	Specify the methods used to collect data from reports, including how many reviewers collected data from each report, whether they worked independently, any processes for obtaining or confirming data from study investigators, and if applicable, details of automation tools used in the process.	Methods, "Data Extraction" (standardized form piloted on two studies; two reviewers independently, discrepancies resolved by consensus); new paragraph explicitly states study authors were not contacted for missing/unclear data.
Data items	10a	List and define all outcomes for which data were sought. Specify whether all results that were compatible with each outcome domain in each study were sought (e.g. for all measures, time points, analyses), and if not, the methods used to decide which results to collect.	Methods, "Eligibility Criteria" (Primary/Secondary Outcomes) plus new sentence: "All outcome measures and timepoints reported by each study were extracted; no results compatible with the outcome domains were excluded."

Section and Topic	Item #	Checklist item	Location where item is reported
	10b	List and define all other variables for which data were sought (e.g. participant and intervention characteristics, funding sources). Describe any assumptions made about any missing or unclear information.	Methods, "Data Extraction" lists variables. A sentence on assumptions for aggregate hospitalization-level data has been started ("For studies reporting only aggregate hospitalization-level data...") but the paragraph is cut off / incomplete in the current draft — needs to be finished.
Study risk of bias assessment	11	Specify the methods used to assess risk of bias in the included studies, including details of the tool(s) used, how many reviewers assessed each study and whether they worked independently, and if applicable, details of automation tools used in the process.	Methods, "Risk of Bias Assessment" (Newcastle-Ottawa Scale; low/moderate/high grading; two reviewers independently).
Effect measures	12	Specify for each outcome the effect measure(s) (e.g. risk ratio, mean difference) used in the synthesis or presentation of results.	Methods, "Synthesis": "Individual study effect estimates (odds ratios, risk ratios, or hazard ratios, as reported by the original authors) were extracted and presented as reported, without conversion to a common effect measure."
Synthesis methods	13a	Describe the processes used to decide which studies were eligible for each synthesis (e.g. tabulating the study intervention characteristics and comparing against the planned groups for each synthesis (item #5)).	Methods, "Synthesis": "Studies were assigned to each synthesis based on whether they reported data for that outcome domain (mortality, length of stay, or complications)."
	13b	Describe any methods required to prepare the data for presentation or synthesis, such as handling of missing summary statistics, or data conversions.	Methods, "Synthesis": "Where summary statistics were incompletely reported or denominators differed across studies (e.g., per-episode vs. per-patient), this is noted alongside the relevant result rather than [converted]." Note: this sentence also appears cut off mid-word ("rather than Due to...") — needs a small copy-edit fix so it reads as one complete sentence.
	13c	Describe any methods used to tabulate or visually display results of individual studies and syntheses.	Tables 1-4 and Fig. 1-2 present results; per current draft, study characteristics and selection/exclusion reasons are displayed in Fig. 1, and individual study findings are tabulated in Table 4.
	13d	Describe any methods used to synthesize results and provide a rationale for the choice(s). If meta-analysis was performed, describe the model(s), method(s) to identify the presence and extent of statistical heterogeneity, and software package(s) used.	Methods, "Synthesis" (narrative synthesis rationale: heterogeneity across populations/settings/outcome definitions; "no pooled estimate was calculated"). Not applicable: no software package or heterogeneity statistic, since no meta-analysis was performed.
	13e	Describe any methods used to explore possible causes of heterogeneity among study results (e.g. subgroup analysis, meta-regression).	Not applicable — no meta-analysis performed; not pre-specified as a narrative-synthesis method.
	13f	Describe any sensitivity analyses conducted to assess robustness of the synthesized results.	Not applicable — no meta-analysis performed.
Reporting bias assessment	14	Describe any methods used to assess risk of bias due to missing results in a synthesis (arising from reporting biases).	Not reported.
Certainty assessment	15	Describe any methods used to assess certainty (or confidence) in the body of evidence for an outcome.	Not reported.
**RESULTS**			

Section and Topic	Item #	Checklist item	Location where item is reported
Study selection	16a	Describe the results of the search and selection process, from the number of records identified in the search to the number of studies included in the review, ideally using a flow diagram.	Reported via Fig. 1 (PRISMA flow diagram), referenced in Methods and Results ("Eight studies were included"). Confirm Fig. 1 itself displays the numeric counts at each stage (identified/screened/excluded/included).
	16b	Cite studies that might appear to meet the inclusion criteria, but which were excluded, and explain why they were excluded.	Reported via Fig. 1, per current draft (exclusion reasons now shown in the flow diagram). Confirm the excluded studies and reasons (e.g., population overlap, PICO non-compliance) are legible/labelled within Fig. 1 itself, since no excluded-study list appears in the body text.
Study characteristics	17	Cite each included study and present its characteristics.	Reported via Fig. 1 and Table 1, and Results "Study characteristics" narrative paragraph.
Risk of bias in studies	18	Present assessments of risk of bias for each included study.	Results — NOS quality assessment statement ("all eight included studies were rated high quality, NOS score ≥7/9"); Fig. 2.
Results of individual studies	19	For all outcomes, present, for each study: (a) summary statistics for each group (where appropriate) and (b) an effect estimate and its precision (e.g. confidence/credible interval), ideally using structured tables or plots.	Reported. Individual-study findings are tabulated in Table 4, and effect estimates with 95% CIs now appear in the narrative (e.g., Allareddy: OR 67.53, 95% CI 34.12–133.63; OR 8.73, 95% CI 2.16–35.24). Spot-check that every effect estimate cited in Results/Discussion — not just Allareddy's — carries a CI, since several mortality percentages (e.g. Cheminet 0.95%, Vichinsky 3%) are still reported without precision estimates.
Results of syntheses	20a	For each synthesis, briefly summarise the characteristics and risk of bias among contributing studies.	Results, "Clinical outcomes" / "Predictors of severe outcomes and mortality," alongside NOS ratings.
	20b	Present results of all statistical syntheses conducted. If meta-analysis was done, present for each the summary estimate and its precision (e.g. confidence/credible interval) and measures of statistical heterogeneity. If comparing groups, describe the direction of the effect.	Not applicable — Methods states "no pooled estimate was calculated."
	20c	Present results of all investigations of possible causes of heterogeneity among study results.	Not applicable — no meta-analysis performed.
	20d	Present results of all sensitivity analyses conducted to assess the robustness of the synthesized results.	Not applicable — no meta-analysis performed.
Reporting biases	21	Present assessments of risk of bias due to missing results (arising from reporting biases) for each synthesis assessed.	Not reported.
Certainty of evidence	22	Present assessments of certainty (or confidence) in the body of evidence for each outcome assessed.	Not reported.
**DISCUSSION**			
Discussion	23a	Provide a general interpretation of the results in the context of other evidence.	Discussion section (subheadings: Mortality Burden; Mechanical Ventilation as a Predictor of Mortality; Predictors of ICU Admission; Pulmonary Hypertension and Cor Pulmonale; Infectious Etiology and Antibiotic Management; Procalcitonin as a Severity Marker; Comorbidities/Genotype; Early-Onset vs Secondary ACS).
	23b	Discuss any limitations of the evidence included in the review.	Reported. New "Limitations" paragraph: heterogeneity across populations/settings/outcome definitions/unit of analysis, predominance of

Section and Topic	Item #	Checklist item	Location where item is reported
			retrospective designs, one administrative/coded dataset subject to misclassification, geographic concentration limiting generalizability.
	23c	Discuss any limitations of the review processes used.	Reported. Following paragraph: narrative-synthesis substitution for pre-specified meta-analysis, six-database search without dedicated grey-literature/trial-registry search, exclusion of conference abstracts without full-text data, and no pre-specified language restriction.
	23d	Discuss implications of the results for practice, policy, and future research.	Conclusion paragraph; "Future recommendation" paragraph.
**OTHER INFORMATION**			
Registration and protocol	24a	Provide registration information for the review, including register name and registration number, or state that the review was not registered.	Abstract and Methods — "prospectively registered on PROSPERO (CRD420261295111)."
	24b	Indicate where the review protocol can be accessed, or state that a protocol was not prepared.	Not reported. No PROSPERO URL or other protocol-access statement is present.
	24c	Describe and explain any amendments to information provided at registration or in the protocol.	Abstract/Methods and the new Limitations paragraph both state meta-analysis "was pre-specified but replaced by narrative synthesis due to substantial clinical and methodological heterogeneity," and explicitly acknowledge this reduces quantitative precision relative to the original protocol. Not reported: a formal amendment date/version number.
Support	25	Describe sources of financial or non-financial support for the review, and the role of the funders or sponsors in the review.	"Funding" statement — no specific grant received.
Competing interests	26	Declare any competing interests of review authors.	"Conflict of Interest" statement — none declared.
Availability of data, code and other materials	27	Report which of the following are publicly available and where they can be found: template data collection forms; data extracted from included studies; data used for all analyses; analytic code; any other materials used in the review.	Not reported.

From: Page MJ, McKenzie JE, Bossuyt PM, Boutron I, Hoffmann TC, Mulrow CD, et al. The PRISMA 2020 statement: an updated guideline for reporting systematic reviews. BMJ 2021;372:n71. doi: 10.1136/bmj.n71. This work is licensed under CC BY 4.0. To view a copy of this license, visit https://creativecommons.org/licenses/by/4.0/
