# Supplementary material for: Mortality Associated with Intensive Care Unit Admission and Mechanical Ventilation in Adults with Acute Chest Syndrome: A Systematic Review
Source: Medicina (Kaunas). 2026 Sep 8;62(9):1731. doi: 10.3390/medicina62091731 (PMC13609638; doi:10.3390/medicina62091731)
Supplement: Supplementary file 1 [file medicina-62-01731-s001.zip › Supplementary Materials File S2.pdf]

Database Search Strategy

Systematic Review: ACS in Adults — ICU, Mechanical Ventilation, and Mortality Outcomes

1. PubMed

```
("Anemia, Sickle Cell"[MeSH] OR "Hemoglobin, Sickle"[MeSH]
 OR "sickle cell disease"[tiab] OR "sickle cell"[tiab]
 OR "sickle-cell disease"[tiab] OR HbSS[tiab] OR HbSC[tiab]
 OR "hemoglobin S"[tiab] OR SCD[tiab])
AND
("Acute Chest Syndrome"[MeSH] OR "acute chest syndrome"[tiab]
 OR "acute chest"[tiab])
AND
("Intensive Care Units"[MeSH] OR "Critical Care"[MeSH]
 OR "Critical Illness"[MeSH]
 OR "intensive care unit"[tiab] OR ICU[tiab]
 OR "critical care"[tiab] OR "critically ill"[tiab])
AND
("Respiration, Artificial"[MeSH] OR "Noninvasive Ventilation"[MeSH]
 OR "mechanical ventilation"[tiab] OR intubation[tiab]
 OR NIV[tiab] OR IMV[tiab] OR HFNC[tiab]
 OR "high flow nasal cannula"[tiab])
AND
("Hospital Mortality"[MeSH] OR "Length of Stay"[MeSH]
 OR mortality[tiab] OR death[tiab] OR "length of stay"[tiab]
 OR LOS[tiab] OR outcome*[tiab])
AND
("Adult"[MeSH] OR adult[tiab] OR adults[tiab])
NOT ("child"[MeSH] OR "infant"[MeSH] OR pediatric[tiab] OR paediatric[tiab])
```

2. Scopus

```
TITLE-ABS-KEY("acute chest syndrome")
AND TITLE-ABS-KEY("sickle cell disease" OR "sickle cell" OR "sickle-cell"
  OR "hemoglobin S" OR HbSS OR HbSC OR SCD)
AND TITLE-ABS-KEY("intensive care unit" OR ICU OR "critical care"
  OR "critically ill" OR "intensive care")
AND TITLE-ABS-KEY("mechanical ventilation" OR "mechanically ventilated"
  OR intubation OR NIV OR IMV OR HFNC)
AND TITLE-ABS-KEY(mortality OR death OR "length of stay" OR LOS OR outcome)
AND TITLE-ABS-KEY(adult OR adults)
AND NOT TITLE-ABS-KEY(child OR children OR pediatric OR paediatric OR infant)
```

3. Web of Science

```
TS=("acute chest syndrome")
AND TS=("sickle cell disease" OR "sickle cell" OR "sickle-cell"
  OR "hemoglobin S" OR HbSS OR HbSC OR SCD)
AND TS=("intensive care unit" OR ICU OR "critical care" OR "critically ill")
AND TS=("mechanical ventilation" OR intubation OR NIV OR IMV OR HFNC)
AND TS=(mortality OR death OR "length of stay" OR LOS OR outcome)
AND TS=(adult OR adults)
NOT TS=(child OR children OR pediatric OR paediatric OR infant)
```

4. Cochrane Library (CENTRAL)

```
(MeSH descriptor: [Anemia, Sickle Cell] explode all trees
 OR "sickle cell disease":ti,ab,kw OR "sickle cell":ti,ab,kw
 OR HbSS:ti,ab,kw OR HbSC:ti,ab,kw OR SCD:ti,ab,kw)
AND
(MeSH descriptor: [Acute Chest Syndrome] explode all trees
 OR "acute chest syndrome":ti,ab,kw OR "acute chest":ti,ab,kw)
AND
(MeSH descriptor: [Intensive Care Units] explode all trees
 OR MeSH descriptor: [Critical Care] explode all trees
 OR "intensive care unit":ti,ab,kw OR ICU:ti,ab,kw
 OR "critical care":ti,ab,kw OR "critically ill":ti,ab,kw)
AND
(MeSH descriptor: [Respiration, Artificial] explode all trees
 OR "mechanical ventilation":ti,ab,kw OR intubation:ti,ab,kw
 OR NIV:ti,ab,kw OR IMV:ti,ab,kw)
AND
(MeSH descriptor: [Hospital Mortality] OR MeSH descriptor: [Length of Stay]
 OR mortality:ti,ab,kw OR death:ti,ab,kw OR "length of stay":ti,ab,kw)
AND
(MeSH descriptor: [Adult] OR adult:ti,ab,kw OR adults:ti,ab,kw)
```

5. EBSCO / CINAHL

```
(MH "Anemia, Sickle Cell+" OR TI "sickle cell disease"
 OR AB "sickle cell disease" OR TI "sickle cell" OR AB "sickle cell"
 OR TI HbSS OR TI HbSC OR TI SCD OR AB SCD)
AND
(TI "acute chest syndrome" OR AB "acute chest syndrome"
 OR TI "acute chest" OR AB "acute chest")
AND
(MH "Intensive Care Units+" OR MH "Critical Care+"
```

```
 OR TI "intensive care unit" OR AB "intensive care unit"
 OR TI ICU OR AB ICU OR TI "critically ill" OR AB "critically ill")
AND
(MH "Ventilation, Mechanical+" OR MH "Noninvasive Ventilation"
 OR TI "mechanical ventilation" OR AB "mechanical ventilation"
 OR TI intubation OR AB intubation OR TI NIV OR TI IMV)
AND
(MH "Hospital Mortality" OR MH "Length of Stay"
 OR TI mortality OR AB mortality OR TI death OR AB death
 OR TI "length of stay" OR AB "length of stay")
AND (MH "Adult" OR TI adult OR AB adult)
NOT (MH "Child" OR MH "Infant" OR TI pediatric OR TI paediatric)
```

Notes

- All searches limited to adults (≥18 years)
- No language restriction applied at search stage.

.
